# Cross-sectional investigation and correlation analysis of psychology of college students returning to campus after COVID-19 lockdown lift

**DOI:** 10.3389/fpsyt.2022.915042

**Published:** 2022-07-22

**Authors:** Zhifeng Wang, Bing Jiang, Xingtong Wang, Yi Niu, Haihong Xue

**Affiliations:** ^1^Department of Physical Education, Xi’an Polytechnic University, Xi’an, China; ^2^Department of General Education, Shandong First Medical University and Shandong Academy of Medical Science, Tai’an, China

**Keywords:** COVID-19 pandemic, lockdown lift, college students, return to campus, mental health, investigation

## Abstract

**Objective:**

To conduct a large cross-sectional survey of the mental health of college students during the recovery period of the COVID-19 epidemic.

**Methods:**

Symptom Checklist 90 (SCL-90) and COVID-19 questionnaire were used to investigate the overall mental health level and cognition of epidemic situation of college students in seven colleges and universities in Shaanxi Province.

**Results:**

(1) In the recovery period of COVID-19 epidemic, college students still had psychological and somatic symptoms such as obsessive-compulsive disorder, interpersonal sensitivity, anxiety, hostility, and poor appetite or insomnia; (2) female college students, science and engineering college students, freshmen and senior graduates, and some ethnic minority college students were all groups with psychological symptoms; (3) the psychological status of college students was related to their perception of COVID-19 epidemic, and the more knowledge about epidemic prevention and control, the more confident they were in overcoming the epidemic, and the milder the psychological symptoms.

**Conclusion:**

College students still have some mental health problems in the recovery period of COVID-19 epidemic, which should be paid attention to by education authorities and colleges and universities.

## Introduction

The outbreak of novel coronavirus pneumonia (COVID-19) at the end of 2019 was another major public health emergency worldwide following the severe acute respiratory syndrome (SARS) in 2003, influenza A (H1N1) 2009, Middle East respiratory syndrome (MERS) outbreak in 2012, and Ebola virus (EVD) outbreak in 2014 (1). While the epidemic situation of major infectious diseases causes the death of a large number of people, it can also cause widespread public psychological crisis problems such as tension, panic, anxiety, and depression (2, 3). Leong Bin Abdullah et al. (4) have reported that, After announcing COVID-19 as a global pandemic, the detection rates of depression, anxiety, and depression with combined anxiety symptoms in the general population were as high as 23.9, 41.7, and 19.9%, respectively, which were much higher than those in the pre-outbreak period. Findings on college students also show that, seventy-one percent of college students felt increased stress and anxiety after the COVID-19 outbreak. In addition to its impact on the mental health of college students, COVID-19 severely reduces the quality of life of college students (5). The results of a cross-sectional survey study of 316 college students showed that, the quality of life of college students after the COVID-19 outbreak was below normal levels in the general population before the pandemic, and this effect continued until after the blockade was lifted (6). According to the spread range and speed of the epidemic in different periods, the number of infected cases, and the different social understanding and coping styles of the epidemic, the epidemic can be divided into three periods: the preparation phase, the punctum maximun phase, and the return to normality phase. The public psychological crisis problems in different periods are not the same, and show significantly different characteristics of phased psychological symptoms (7).

The impact of the COVID-19 epidemic on the mental health of the general public (8, 9), medical staff (10, 11), college students (5), isolated individuals and patients (12, 13) has been concerned by many scholars, but these studies come from the preparation phase and the punctum maximun phase, and investigations on the public psychology of the recovery phase of the epidemic are still very rare. Literature suggests that mental health symptoms will outlast the acute phase of the pandemic, and some psychological and somatic symptoms will even last for a lifetime (14–16). Studies of public psychological problems associated with SARS and EVD outbreaks have found that 27.5–83.3% of people experience significant anxiety symptoms even after the outbreak (17, 18), and 12–75% are diagnosed with depression (19, 20). A recent survey of the mental health of college students associated with the COVID-19 epidemic also showed that the detection rates of mild to severe depression, anxiety, and stress in college students were as high as 9.2–15.5%, 7.0–13.2%, and 9.5–26.3% even after the blockade was relieved (21). Thus, the problem of public psychological crisis caused by the epidemic does not disappear with the end of the epidemic, on the contrary, it lasts for a long time after the end of the epidemic.

College students are a social group in which mental health is highly vulnerable, and they are highly susceptible to psychological crisis problems based on various misperceptions. Presented in the 2019 annual report of the University Mental Health Center, among 82,685 interviewed college students, 62.7% of respondents reported varying degrees of anxiety problems (22), clinicians also reported that anxiety remained the most common diagnostic symptom for college students seeking services at psychological counseling centers. Others have reported that approximately 3–7% of college students still have suicidal thoughts even in the absence of an outbreak, and that college student anxiety is closely related to suicidal behavior (23, 24). During the preparation phase and the punctum maximun phase of the COVID-19 outbreak, more than 150 countries around the world, including China, have closed schools and educational institutions, and the outbreak directly affects more than 80% of students in the world (25). Numerous findings have shown that during the COVID-19 outbreak, college students generally experience a variety of psychological crisis problems such as tension, anxiety, depression and depression (5, 13, 26). It can be concluded that the COVID-19 epidemic is interlaced with the rising mental health problems of college students (27, 28), further increasing the extreme urgency of carrying out mental health research among college students.

At present, the research on the mental health problems of college students during the COVID-19 epidemic mainly focuses on the epidemic preparation period and the comprehensive outbreak period, and further research is needed for the mental health problems of college students during the recovery period. In the recovery period of the COVID-19 epidemic, although college students have returned to school to start learning and life, but because the epidemic has not been completely ended, college students still face the risk of mass infection. In reality, COVID-19 outbreaks occur frequently in colleges and universities, which have caused some impact on the mental health of convalescent college students. How to do a good job in the mental health detection and intervention of college students while adhering to the epidemic prevention and control policies of all regions and maintaining normal teaching activities is a realistic problem facing the education authorities at all levels during the recovery period of the epidemic.

In this study, we investigated and analyzed the mental health status and related influencing factors of college students in seven colleges and universities in China during the recovery period of the epidemic, and proposed psychological interventions for reference. The results of the study are of great practical significance for the government and education departments to comprehensively master the mental health status and various influencing factors of college students in the recovery period of the epidemic, especially after returning to school, and to formulate psychological intervention strategies for college students in line with the current epidemic prevention and control measures and the law of normal teaching activities in colleges and universities.

## Participants and methods

### Participants

**FIGURE 1:**
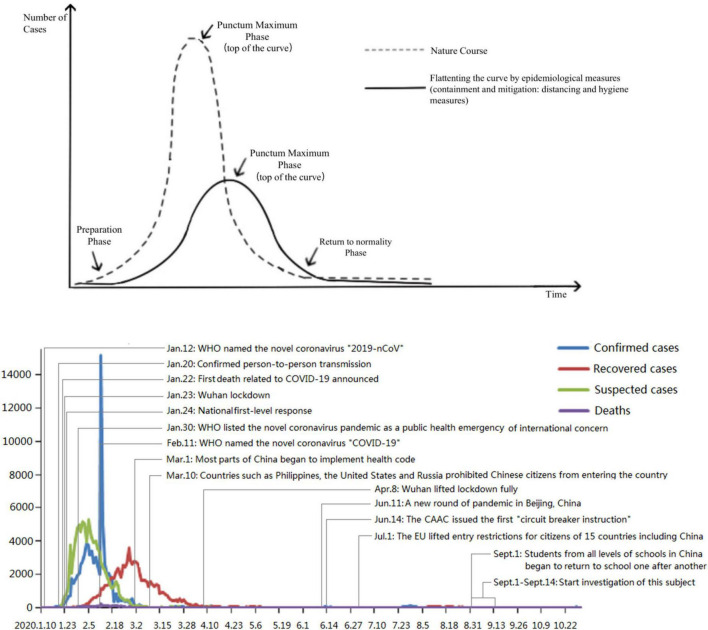
The development trend and major events of the COVID-19 pandemic in China. Statistics were obtained from the official website of National Health Commission of the People’s Republic of China.

In the form of mobile phone questionnaire star, a comprehensive survey was conducted among seven college students in Shaanxi Province. The questionnaire was distributed from September 1 to 14, 2020. A total of 2,969 questionnaires were obtained and ineffective questionnaires were excluded, and a total of 2,642 valid questionnaires were collected, with a questionnaire response rate of 88.99%. A valid questionnaire is not completed with all options for each questionnaire and is considered a valid questionnaire as long as its data are valid when a local question analysis is done. Exclusion criteria for invalid questionnaires: Answering time less than 5 min or the presence of a large number of missed options or excessive similar answers in the questionnaire. From the outbreak of pneumonia in Xinguan to the investigation of this topic, the overall development trend of the epidemic in China is shown in Figure 1, and the basic demographic information of the students participating in the investigation of this topic (mean age 22.54 ± 5.64 years) is shown in Table 1.

**TABLE 1:** Demographic distribution of effective sample size in this study.

		Recycle (portion)	Valid (portion)	Efficient (%)
**Gender**				
	M	1456	1236	84.89
	F	1513	1406	92.93
**Grade**				
	Freshman	929	818	88.06
	Sophomoreer	767	678	88.40
	Junior	547	491	89.76
	Senior	507	457	90.14
	Postgraduate	219	198	90.82
**Major**				
	Literature and history	802	714	89.03
	Science and engineering	931	867	93.13
	Art	738	616	83.52
	Sports	498	445	89.26
**Nationality**				
	Han	1994	1777	89.11
	Hui	326	298	94.41
	Man	231	205	88.74
	Uygur	130	111	85.38
	Mongol	114	106	92.98
	Others	174	145	83.33
**Region**				
	Hubei	487	433	88.91
	Shaanxi	1113	1005	90.30
	Others	1369	1204	87.95

Other ethnic groups include Tujia, Xibo, Tibetan, Kirgiz, Kazakh, Miao, etc.; other provinces and autonomous regions include: Henan, Shandong, Jiangsu, Liaoning, Heilongjiang, Xinjiang, Tibet, Gansu, etc.

### Tools

#### Demographic questionnaire

A self-compiled questionnaire was used to investigate the general demographic data of college students: gender, age, major, grade and origin, etc.

#### Symptom checklist 90

The scale was compiled by Derogatis et al. (29), including 90 items, divided into 10 dimensions, namely somatization (12 items), compulsion (10 items), interpersonal sensitivity (9 items), depression (13 items), anxiety (10 items), hostility (6 items), terror (7 items), paranoid (6 items), psychoticism (10 items) and other dimensions to assess disturbances in appetite and sleep (7 items). In order to evaluate the mental symptoms of college students more effectively, we used the adaptation scale revised by Tang et al. (30), which had been proved to have high reliability and validity in Chinese population with a Cronbach’s alpha coefficient of 0.96. At present, this scale has been widely used in psychological symptom evaluation of college students (31, 32).

According to the literature (33), the average of all 90 items in symptom checklist 90 (SCL-90) was taken as the global severity index (GSI) in this study. For convenience, the raw GSI scores were converted into T scores (mean = 50, *SD* = 10) (7), and used to assess the severity of the overall psychological symptoms (34).

#### The COVID-19 pandemic cognitive questionnaire

According to the “Novel coronavirus transmission routes and prevention guidelines” and “2019 novel coronavirus pandemic development and risk assessment” and related literature issued by Chinese Center for Disease Control and Prevention on January 28, 2020, the COVID-19 pandemic cognitive questionnaire was compiled (35). The scale was divided into four dimensions, namely the basic knowledge of COVID-19, the confidence in curing COVID-19, the overall cognitive mentality in the face of COVID-19 and the satisfaction with epidemic prevention and control measures, with a total of 14 items. To test the reliability and validity of the questionnaire, this study performed two repeated pretests before formal testing, with a 2-week interval between repeated tests. The questionnaire was distributed among 200 college students at the time of the prediction test. The statistical software SPSS 23.0 was used for descriptive statistics, correlation analysis, item analysis, exploratory factor analysis and reliability analysis of the questionnaire results, and AMOS23.0 was used for confirmatory factor analysis. The results showed that the overall Cronbach’s α coefficient of the questionnaire was 0.908.

### Statistics

SPSS 23.0 statistical software (IBM SPSS statistics, New York, United States) was used for descriptive analysis, correlation analysis, one-way analysis of variance (ANOVA), multiple linear regression analysis and *t*-test etc. Kolmogorov-Smirnov was used to test the normality of the sample data (*p*> 0.05 means normal distribution). All measurement data were expressed as mean ± standard deviation ($\bar{x}$ ± s), and count data were expressed as percentage (%). The significant level was *p* < 0.05, and the extremely significant level was *p* < 0.01.

## Results

### Demographic characteristics of participate

Among the valid questionnaires returned, females accounted for a slightly higher proportion (1406/2642, 53.22%) than males (1236/2642, 46.78%). The proportion of freshman students was the highest (30.96%), the proportion of graduate students was the lowest (7.49%), and the proportion of students in other grades was in between. From the professional distribution, science and engineering students accounted for the highest proportion (32.81%), and the lowest proportion was sports students (16.84). From the ethnic distribution, Han students were the highest among the sampled students (67.30%), followed by Hui (11.30%), Manchu (7.76%), Uighur (4.2%), and Mongolian (4.01%), and other ethnic minority students accounted for a relatively small proportion. From the regional distribution of the sample, Shaanxi students accounted for the highest proportion (38.4%). From the overall demographic distribution characteristics of the sample, the effective sample distribution in this study basically conforms to the ethnic structure characteristics of the Chinese population and the nature of the sampled schools.

### Comparison of symptom checklist 90 scores with the norms of Chinese college students in the phase of return to normality of the COVID-19 pandemic

The results in Table 2 showed that, compared with the norm of Chinese college students (36), the scores of compulsion, interpersonal sensitivity, anxiety, depression, hostility and terror increased extremely significantly (*p* < 0.01 or *p* < 0.05), the scores of somatization, paranoid and psychoticism had a upward trend (*p* > 0.05). The results suggested that the overall mental health of college students in the phase of return to normality of the COVID-19 pandemic was still in a low state.

**TABLE 2:** Comparison of SCL-90 scores with the norms of Chinese college students ($\bar{x}$ ± s).

Factors	Participant (n = 2642)	Norm of Chinese college students (*n* = 4141)	*t*	*P*
Somatization	1.54 ± 0.42	1.45 ± 0.49	0.96	0.30
Compulsion	2.58 ± 0.81	1.98 ± 0.64	20.91	0.00
Interpersonal sensitivity	2.45 ± 0.77	1.98 ± 0.74	17.23	0.00
Depression	1.97 ± 0.58	1.83 ± 0.65	3.34	0.04
Anxiety	2.29 ± 0.69	1.64 ± 0.59	15.54	0.00
Hostility	2.08 ± 0.74	1.77 ± 0.68	13.28	0.00
Terror	1.82 ± 0.57	1.46 ± 0.53	7.78	0.00
Paranoid	1.87 ± 0.74	1.85 ± 0.69	0.88	0.34
Psychoticism	1.71 ± 0.64	1.63 ± 0.54	1.17	0.14
Other	2.18 ± 0.61	–	–	–

The results in Table 3 showed that the average of the ten dimensions of SCL-90 was between 1.54 and 2.58, among which anxiety, interpersonal sensitivity, compulsion, hostility, sleep and appetite were all positive. The results suggested that in the phase of return to normality of the COVID-19 pandemic, even if the pandemic had been effectively controlled and social production and life had gradually recovered, the impact of the pandemic on public psychology still existed, and college students still had compulsion, interpersonal sensitivity, anxiety, hostility, poor appetite, insomnia and other psychological and physical symptoms.

**TABLE 3:** Distribution of SCL-90 scores in all dimensions (n, %).

Factors		1	1 < i < 2		2 < i < 3		3 < i < 4		4 < i < 5	
Somatization	2229	84.36%	207	7.83%	139	5.26%	44	1.66%	24	0.89%
Compulsion	1638	61.98%	522	19.74%	331	12.51%	125	4.74%	27	1.03%
Interpersonal sensitivity	1525	57.72%	529	20.04%	373	14.12%	183	6.93%	31	1.19%
Depression	1561	59.08%	562	21.27%	323	12.24%	166	6.29%	30	1.12%
Anxiety	1347	50.99%	642	24.31%	403	15.27%	216	8.16%	33	1.26%
Hostility	1776	67.21%	355	13.42%	294	11.11%	193	7.29%	25	0.95%
Terror	2351	88.98%	153	5.79%	85	3.21%	32	1.22%	21	0.78%
Paranoid	2332	88.27%	180	6.83%	111	4.22%	56	2.11%	15	0.57%
Psychoticism	2423	91.70%	138	5.21%	40	1.50%	28	1.05%	14	0.54%
Other	1677	63.49%	467	17.67%	294	11.14%	167	6.33%	36	1.36%

i refers to the dimension score.

### Comparison of the global psychological severity index of college students in demographic variables

GSI T-score was the average of all 90 items in SCL-90, and it had been used as an important indicator to evaluate the severity of group psychological symptoms (34). In this study, one-way ANOVA was used to compare the differences of GSI T-scores in demographic variables. Before the analysis of variance, we used the Levene test to examine the homogeneity of variance.

As shown in Table 4, in Levene’s test for homogeneity of variance, the variances of the sample data between genders were uniform (*p* = 0.867). In Kolmogorov- Smirnov test, the data of each group followed the normal distribution (*p > 0.05*). The results of one-way ANOVA showed that there was a significant difference in GSI T-score between male and female college students (*F* = 3.435, *p* = 0.032).

**TABLE 4:** Comparison of GSI T-score between different genders.

Gender	Number	Mean ± SD	N	%	Homogeneity of variance test	F
Male	1	40.36 ± 2.59	1236	46.78	0.867	3.435*
Female	2	45.36 ± 3.57	1406	53.22		

*P < 0.05.

As shown in Table 5, in Levene’s test for homogeneity of variance, the variances of data among different majors were inconsistent (*p* = 0.002), and the data of each group followed the normal distribution in Kolmogorov-Smirnov test (*p* > 0.05). The results of one-way ANOVA showed that there were extremely significant statistical significances in GSI T-scores among college students of different majors (*F* = 8.314, *p* < 0.01). The order of GSI T-scores among majors was science and engineering, literature and history, art and sports.

**TABLE 5:** Comparison of GSI T-score among different majors.

Majors	Number	Mean ± SD	N	%	Homogeneity of variance test	*F*
Literature and history	1	45.35 ± 3.01	714	27.02	0.002	8.314**
Science and engineering	2	47.86 ± 3.65	867	32.82		2 > 1 > 3 > 4
Art	3	41.24 ± 2.81	616	23.32		
Sports	4	40.86 ± 2.07	445	16.84		

**P < 0.01.

As shown in Table 6, in Levene’s test for homogeneity of variance, the variances of data among different grades and postgraduates were inconsistent (*p* = 0.003), in Kolmogorov-Smirnov test, the data of each group followed the normal distribution (*p* > 0.05). The results of one-way ANOVA showed that GSI T-scores were extremely statistical significant among different grades and postgraduates (*F* = 6.574, *p* < 0.01), and the order of GSI T-score was freshmen, senior, sophomore, junior and postgraduate.

**TABLE 6:** Comparison of GSI T-score among different grades.

Grades	Number	Mean ± SD	N	%	Homogeneity of variance test	*F*
Freshmen	1	51.55 ± 4.86	818	30.96	0.003	6.574**
Sophomore	2	45.89 ± 5.69	678	25.66		1 > 4 > 2 > 3 > 5
Junior	3	40.28 ± 3.88	491	18.58		
Senior	4	48.75 ± 5.14	457	17.31		
Postgraduate	5	38.87 ± 4.41	198	7.49		

**P < 0.01.

As shown in Table 7, in Levene’s test for homogeneity of variance, the variances of data among different regions were inconsistent significantly (*p* = 0.001), and the data of each group followed the normal distribution in Kolmogorov-Smirnov test (*p* > 0.05). The results of one-way ANOVA showed that there were extremely significant statistical differences in GSI T-scores among college students in different regions (*F* = 6.892, *p* < 0.01), the order of GSI T-score was Hubei, other regions and Shanxi.

**TABLE 7:** Comparison of GSI T-score among different regions.

Regions	Number	Mean ± SD	N	%	Homogeneity of variance test	*F*
Hubei	1	50.92 ± 6.62	433	16.39	0.001	6.892**
Shanxi	2	42.89 ± 5.75	1005	38.04		1 > 3 > 2
Other	3	43.71 ± 4.80	1204	45.57		

**P < 0.01.

As shown in Table 8, in Levene’s test for homogeneity of variance, the variances of data among different nationalities were in significantly consistent (*p* = 0.001), and the data of each group followed the normal distribution in Kolmogorov-Smirnov test (*p* > 0.05). The results of one-way ANOVA showed that there were extremely significant statistical significances in GSI T-scores among college students in different nationalities (*F* = 9.129, *p* < 0.01), the order of GSI T-score was Uighur, Hui, Han, Mongolian, other nationality and Manchu.

**TABLE 8:** Comparison of GSI T-score among different nationalities.

Nationalities	Number	Mean ± SD	N	%	Homogeneity of variance test	*F*
Han	1	45.45 ± 4.67	1777	67.26	0.001	9.129**
Hui	2	50.81 ± 5.63	298	11.28		4 > 2 > 1 > 5 > 6 > 3
Manchu	3	40.39 ± 4.29	205	7.76		
Uighur	4	51.81 ± 5.65	111	4.20		
Mongolian	5	42.76 ± 3.69	106	4.01		
Other	6	41.99 ± 4.85	145	5.49		

**P < 0.01.

### Correlation analysis between the cognition of the COVID-19 pandemic and global severity index T-score

The results of multiple regression analysis in Table 9 showed that the more they knew about the danger of COVID-19, the lower the GSI T-score (β = –4.521, –2.632, –0.354, all *p* < 0.01); The more they knew about the infectivity of COVID-19, the lower the GSI T-score (β = −3.524, –1.341, –0.125, *p* < 0.01 or *p* < 0.05); The more knowledge about the prevention of COVID-19, the lower the GSI T-score (β = −5.254, –3.241, –0.658, all *p* < 0.01); The more knowledge about the treatment of COVID-19, the lower the GSI T-score (β = −3.587, –1.608, –0.489, all *p* < 0.01); The more they knew about the prognosis rehabilitation of COVID-19, the lower the GSI T-score (β = −3.081, –1.009, –0.389, all *p* < 0.01).

**TABLE 9:** Multiple regression analysis affecting the psychological state of college students.

Variances	GSI T-score		
	
	β	SE	95%CI
**Basic knowledge of COVID-19**			
**Danger to COVID-19 (reference: unknown)**			
Know well	–4.521**	0.421	(–4.611, –3.360)
Know a little	–2.623**	0.312	(–2.352, –1.218)
Know little	–0.354**	0.123	(–0.666, –0.173)
**Infectivity to COVID-19 (reference: unknown)**			
Know well	–3.524**	0.342	(–3.561, –2.269)
Know a little	–1.341**	0.208	(–2.153, –1.313)
Know little	–0.125*	0.151	(–0.756, –0.311)
**Prevention knowledge to COVID-19 (reference: unknown)**			
Know well	–5.254**	0.624	(–5.105, –4.263)
Know a little	–3.241**	0.426	(–3.353, –2.367)
Know little	–0.658**	0.148	(–0.856, –0.213)
**Treatment for COVID-19 (reference: unknown)**			
Know well	–3.587**	0.405	(–3.861, –2.269)
Know a little	–1.608**	0.304	(–1.858, –0.719)
Know little	–0.489**	0.201	(–0.458, –0.091)
**Prognostic rehabilitation of COVID-19 (reference: unknown)**			
Know well	–3.081**	0.501	(–3.163, –2.369)
Know a little	–1.009**	0.324	(–1.059, –0.318)
Know little	–0.389**	0.211	(–0.356, –0.091)
**Awareness of the danger of COVID-19**			
**Unintentional exposure to COVID-19 (reference: unafraid)**			
Afraid extremely	6.082**	0.684	(5.262, 6.161)
Afraid much	4.048**	0.369	(3.717, 4.353)
Afraid a little	1.380**	0.324	(0.819, 1.656)
Going out on business (reference: unafraid)			
Afraid extremely	4.428**	0.504	(3.664, 4.561)
Afraid much	2.855**	0.426	(1.217, 2.750)
Afraid a little	0.181*	0.221	(0.078, 0.391)
**Talking about COVID-19 (reference: unafraid)**			
Afraid extremely	6.781**	0.726	(5.961, 6.868)
Afraid much	4.454**	0.624	(3.711, 4.350)
Afraid a little	0.179*	0.124	(0.009, 0.196)
**Beliefs in COVID-19**			
**Current attitude to COVID-19 (reference: not terrible)**			
More terrible	5.987**	0.620	(4.966, 5.869)
Terrible	4.559**	0.524	(3.718, 4.657)
Terrible a little	0.889**	0.132	(0.076, 0.826)
**Belief in defeating COVID-19 (convinced)**			
Unconvinced	6.887**	0.426	(5.667, 6.569)
Unpredictable	4.952**	0.329	(3.418, 4.356)
Skeptical	3.180**	0.238	(2.209, 3.396)
**Views on COVID-19 (reference: a good thing)**			
A bad thing	4.782**	0.521	(3.968, 4.807)
Neither	0.208*	0.329	(0.012, 0.358)
Both	0.109	0.401	(–0.409, 1.096)
**Awareness of anti-pandemic measures**			
**Social anti-pandemic measures (reference: satisfied)**			
Very dissatisfied	5.724**	0.429	(4.350, 5.321)
Dissatisfied	3.752**	0.331	(2.319, 3.855)
Generally satisfied	1.082**	0.132	(0.943, 1.695)
**Pandemic prevention measures in schools (reference: satisfied)**			
Very dissatisfied	3.983**	0.405	(2.269, 3.861)
Dissatisfied	1.111**	0.365	(0.619, 1.350)
Generally satisfied	0.281**	0.112	(0.091, 0.350)
1 **Family’s pandemic prevention measures (reference: satisfied)**			
Very dissatisfied	4.816**	0.529	(5.660, 6.511)
Dissatisfied	2.958**	0.222	(2.018, 3.358)
Generally satisfied	0.106	0.238	(–0.609, 1.191)

*P < 0.05; **P < 0.01.

Compared with college students who felt unafraid of COVID-19, the more afraid of unintentional exposure to COVID-19, the higher the GSI T-score (β = 1.380, 4.048, 6.082, all *p* < 0.01); The more afraid of going out on business, the higher the GSI T-score (β = 0.181, 2.855, 4.428, all *p < 0.01*); The more afraid of talking about COVID-19, the higher the GSI T-score (β = 0.179, 4.454, 6.781, *p* < 0.05 or *p* < 0.01).

In terms of the overall attitude toward COVID-19, compared with college students who didn’t feel terrible, the more terrible they felt about COVID-19 currently, the higher the GSI T-score (β = 0.889, 4.559, 5.987, all *p* < 0.01); The lower the confidence in overcoming the pandemic, the higher the GSI T-score (β = 3.180, 4.952, 6.887, all *p < 0.01*); Compared with those college students who considered COVID-19 “a good thing,” college students who considered COVID-19 “neither a good thing nor a bad thing” and “a bad thing” had higher GSI T-score (β = 0.208, 4.782, *p* < 0.05 and *p* < 0.01).

In terms of awareness of pandemic prevention measures, compared with those college students who were “satisfied” with social anti-pandemic measures, college students who were dissatisfied with social anti-pandemic measures showed higher GSI T-score (β = 5.724, 3.752, 1.082, all *p* < 0.01). The more dissatisfied with the school’s pandemic prevention measures, the higher the GSI T-score (β = 3.983, 1.111, 0.281, all *p* < 0.01); The more dissatisfied with the family’s pandemic prevention measures, the higher the GSI T-score (β = 3.983, 1.111, 0.281, all *p* < 0.01).

In summary, there was a significant correlation between the cognitive attitude of college students to COVID-19 and psychological symptoms. The more optimistic college students could be about COVID-19, the more they knew about virus protection and treatment, the more satisfied with the epidemic prevention measures of society, schools and families, the lower the GSI T-score, and the better their overall mental health.

## Discussion

### General psychological characteristics of college students in the phase of return to normality of the COVID-19 pandemic

In the phases of preparation and punctum maximum of the COVID-19 pandemic, in order to curb the spread of the pandemic and reduce personnel infection as soon as possible, the Chinese government had decisively postponed the return of college students nationwide in the spring semester of 2020 (in March), and all colleges and universities implemented online teaching. This study was a large-scale cross-sectional survey on the mental health problems of college students returning to school in the phase of return to normality of the pandemic in China in September 2020. The results showed that, compared with SCL-90 norm of Chinese college students in 1998, the scores of compulsion, interpersonal sensitivity, anxiety, hostility, depression and other dimensions in SCL-90 of Chinese college students were significantly or extremely significantly increased in the phase of return to normality of the COVID-19 pandemic (*p* < 0.05 or *p* < 0.01), among which, the average scores of compulsion, interpersonal sensitivity, anxiety, hostility and other dimensions all reached or higher than 2 points, showing obvious positive symptoms, and the dimension of depression was weakly positive. From the perspective of the severity of psychological symptoms, compulsion, interpersonal sensitivity, anxiety and hostility were the four dimensions that had the largest differences from the norms of SCL-90 developed in 1998, and these four dimensions were all positive, indicating that the main psychological symptoms of Chinese college students were compulsion, interpersonal sensitivity, anxiety and hostility in the phase of return to normality of the pandemic. Findings were generally consistent with those reported by Woon et al. (21), who also found higher detection rates of mild to severe depression, anxiety, and stress among university students even after the blockade was lifted. However, just from the detection rate of anxiety symptoms, the detection rate of anxiety symptoms in college students in this study was much higher than that in Cao et al. (37). The results of the study, however, were lower than those reported by Son et al. (5), and the reason for the difference may be related to the different respondents and scales used.

Among the 10 sub-items in the dimension of compulsion, except for item 51 (your brain became empty) and item 28 (felt difficult to complete the task), whose average score did not reach 2 points, the total average score of the other 8 sub-items all reached or exceeded 2 points, showing obviously positive. The results suggested that, in the phase of return to normality of the pandemic, the compulsive symptoms of college students returning to school were mainly focused on repeated unnecessary thoughts in their mind, repeated checks in doing things, feeling difficult to complete tasks, repeated hand washing, and forgetfulness, etc., which might be related to the pandemic prevention measures and personal hygiene habits required by government departments to be observed during the pandemic. Among the nine sub-items in the dimension of interpersonal sensitivity, except for item 41 (feeling inferior to others) and item 73 (feeling uncomfortable eating in public), the total average score of the other seven sub-items all reached or exceeded 2 points, showing obviously positive. The results suggested that, in the phase of return to normality of the pandemic, the interpersonal sensitivity of Chinese college students was mainly manifested in feelings vulnerable, feeling incomprehensible, feeling being treated unfriendly and hypersensitivity to others, etc., and this might be due to the college students returning to school had a certain sense of strangeness after nearly half a year of home isolation; it might also be related to the distrust or suspicion of whether others were virus carriers since the pandemic had not completely ended. Among the 10 sub-items in the dimension of anxiety, seven items had positive scores. The anxiety of college students was manifested as feeling bursts of fear or panic, sudden fear for no reason, nervousness and restlessness, etc., and this might be because college students still felt uncertain about whether the pandemic would make a comeback. Among the six sub-items in the dimension of hostility, except for item 74 (after arguing with others), all the other five sub-items were positive, mainly manifested in uncontrollable tantrum, getting annoyed and excited easily, impulse to hit or hurt others, etc. In summary, even though there had not been a single case of infection among college students in China since the outbreak of the pandemic, college students still showed obvious symptoms of compulsion, interpersonal sensitivity, anxiety and hostility in the phase of return to normality of the pandemic, indicating that the major impact of the pandemic on the lives and studies of college students remained exist.

### Demographic differences in the psychological symptoms of college students in the phase of return to normality of COVID-19

GSI was the average of all 90 items in SCL-90, which could reflect the overall severity of psychological symptoms (34). In order to facilitate the comparison of the differences in the severity of overall psychological symptoms of college students in demographic variables, the GSI of college students in different genders, nationalities, grades, majors and regions was compared in this study. The results showed that the GSI of female college students was significantly higher than that of male college students, and the results of the study were basically consistent with those of Carmassi et al. (38). Under general circumstances, women had delicate emotions and strong sensitivity, and even during non-pandemic periods, the proportion of psychological crisis in woman was larger than that in men (39). In major emergencies, women had stronger psychological symptoms than men (40). Therefore, in this study, the higher GSI of female college students might be related to the stronger mental and psychological stimulation of the pandemic to female college students.

The comparison among majors showed that the GSI of science and engineering college students was higher. Under normal circumstances, long-term learning in a major might have a certain impact on personal psychology and character. The science and engineering courses generally had the characteristics of logic, hierarchy and technology. Long-term study of science and engineering could easily make people develop a rigorous, meticulous or almost stereotyped thinking, resulting in their lower self-emotion regulation and adaptive coping skills and increased maladjustment when facing the major emergency of COVID-19 (41), which in turn led to more serious psychological symptoms.

The comparison among different grades showed that freshmen and seniors had higher GSI. In general, the level of personal education was related to the level of mental health under stress, and a lower level of education often indicated a poorer level of mental health in emergencies (42). Assari also reported that people’s psychological symptoms decrease with the increase of education degree, which was basically consistent with the results of this study (43). Junior students often meant insufficient reserves of professional knowledge and lack of effective coping style. Therefore, they were more prone to various mental health problems in the face of emergencies (44). However, this study also found that the seniors also had higher GSI, which might be due to the fact that they were facing graduation and employment. The uncertain pandemic situation not only affected their successful graduation, but also deteriorated the social employment environment. Both of them aggravated the psychological symptoms of senior graduates.

The comparison among regions showed that the GSI of college students in Hubei was higher. Wuhan, Hubei Province, China, as the epicenter of the COVID-19 pandemic, was the first to be closed and was also the most severely affected by the pandemic. The “stigmatization” of the people in Hubei occurred from time to time after the full outbreak of the pandemic. Some college students from pandemic outbroke area (Wuhan, Hubei) might also worry about being stigmatized or discriminated against from other students and thus bore huge psychological pressure, which further aggravated the original psychological crisis, and this was basically consistent with the results reported in the literature (45).

The comparison among nationalities showed that the GSI of Uighur and Hui college students was relatively higher, and Han was at the medium level. In China, Han was the main ethnic group, accounting for 91.51% of the national population, and other ethnic groups were called ethnic minorities. At present, the results of studies on the mental health problems of ethnic minorities were inconsistent. On the one hand, it was considered that ethnic minorities were poor in social employment, access to medical and health services, and had greater pressure in life. On the other hand, it was also found that the health status of ethnic minorities was no worse than that of the main ethnic groups (46). During the COVID-19 pandemic, the number of cases and deaths of ethnic minorities (African Americans, Latin Americans, American Indians, Alaska Natives and Pacific Islanders) was higher in the United States and Britain (47, 48), which would have immeasurable impact on the mental health of the local minority people. In China, there was no comparative study on the number of infection cases among ethnic groups. However, this study found that Uighur and Hui college students had higher GSI, which might be related to religious beliefs. Studies had found that religious beliefs could increase or decrease people’s psychological symptoms under certain circumstances (49). Both the Uighur and Hui people in China believed in Islam. The special ethnic religious beliefs might limit the interpersonal communication and social support of these groups during the pandemic, which might aggravate the psychological symptoms of ethnic college students. However, the GSI of college students from other ethnic minorities was lower, which might be related to the fact that most of these ethnic groups were located in border areas and were less affected by the pandemic, or it might be related to specific ethnic beliefs.

### Correlation between psychological symptoms and pandemic cognition of college students

This study found that the more college students knew about COVID-19, the lower their fear of the danger of COVID-19, the stronger their confidence in overcoming the pandemic, the more satisfied they were with the anti-pandemic measures of society, schools and families, the lower their GSI, and the higher their mental health level. The results of the study were basically consistent with those of Wang et al. (35). In general, the more knew about COVID-19, the stronger the adaptability and coping ability during the pandemic, the stronger the sense of self-efficacy, and the lower the possibility of psychological crisis (50). The more satisfied with the anti-pandemic measures of society, schools and families, the greater the sense of security and the ability to resist psychological risks. These results provided ideas for the formulation of psychological intervention policies for college students in the phase of return to normality of the pandemic.

## Conclusion

In the phase of return to normality of the COVID-19 pandemic, the mental health of college students had not fully recovered as the pandemic slowed down, and the main psychological or physical symptoms were compulsion, inter personal sensitivity, anxiety, hostility, poor appetite or insomnia.

In the phase of return to normality of the COVID-19 pandemic, the mental health status of college students was different in demographic variables. Female college students, Hubei college students, freshmen, seniors and individual minority (Uighur, Hui) college students had higher GSI scores, suggesting that these college students were all high-risk groups of psychological crisis and needed to be paid more attention.

In the phase of return to normality of the COVID-19 pandemic, the mental health level of college students was related to their awareness of the pandemic. The more college students knew about the pandemic prevention and control, the stronger their confidence in overcoming the pandemic, the more satisfied they were with the prevention measures of society, schools and families, and the more calm they were in the face of the pandemic threat, the higher their mental health level, and vice versa.

### Suggestion on mental health intervention for college students in the phase of return to normality of the pandemic

#### Government education departments and universities should pay attention to the remaining problems of the pandemic

The slowdown of the pandemic did not mean the disappearance of the problems left over by the pandemic. At present, the psychological crisis of college students has become one of the important legacy issues of the COVID-19 pandemic, which should arouse great attention from government education departments and universities. This requires government education departments and universities to fully understand the long-term and complexity of college students’ mental health problems, and pay continuous attention to the mental health problems of college students in the phase of return to normality of the pandemic, especially the key groups such as women, junior, graduates and individual minority students. We should regularly test and evaluate the psychological conditions of these groups and provide targeted psychological rescue or intervention services in time. Targeted psychological intervention can be provided through multiple channels such as offline cognitive behavior intervention, sports, WeChat, Internet and telephone. Secondly, we should provide more information on the pandemic prevention and control for key high-risk groups, dissolve the fear of the pandemic, boost the confidence in overcoming the pandemic, and improve psychological self-adjustment ability.

#### Strengthen the screening of high-risk groups of psychological crisis, and establish a long-term detection and intervention mechanism

After studying the public psychological crisis during the SARS in 2003, H1N1 in 2009, MERS in 2012 and EVD in 2014, it was found that different individuals had significantly different psychological responses when facing the same pandemic threat. Some individuals had serious psychological stress response in the face of the pandemic, resulting in psychological crisis problems such as anxiety, depression and panic, which lasted for a long time. However, some individuals only showed slight psychological stress response during the pandemic, and the response would disappear quickly or heal on its own. There was a problem of vulnerability. Some individuals with strong vulnerability would produce strong negative emotional response even in the face of neutral or slight stress stimulation, and evolve into serious psychological crisis, which would last for a long time. However, individuals with weaker vulnerability could effectively cope with strong stress stimulation, and their psychological stress response was weaker and the duration was shorter (1). The results of neurobiology and epidemiology studies provided support for the issue of vulnerability among individuals. Studies had shown that some factors could increase the risk of bad mood in adulthood, such as small natural hippocampus (51), BDNF gene polymorphism (52), abnormal expression of serotonin 2A receptor gene (HRT2A) (53) and tryptophan hydroxylase 2 gene (TPH2) (54), small weight at birth (55), abuse in childhood, loss of parents, malnutrition and discordant family environment (56, 57). Therefore, it is necessary to increase the investigation of the above-mentioned factors among college students, establish data on high-risk groups, and provide targeted intervention to reduce the psychological vulnerability of these groups, which can effectively improve the psychological adaptability of these groups in the new public crisis in the future.

#### The shortcomings of this study

This study is a large-scale cross-sectional survey of the mental health of college students in the recovery period of the COVID-19 epidemic in China, which not only analyzes the overall mental health status and demographic distribution characteristics of college students in the recovery period of the epidemic, but also discusses the relevant influencing factors from the perspective of cognitive psychology, and finally proposes the relevant interventions and preventive measures for the mental health of college students in the recovery period of the epidemic. The results of this study are important for education authorities to comprehensively master the mental health status and related influencing factors of college students in the recovery period of the epidemic and formulate intervention strategies for phase. This is both the difference between this study and other studies and the bright spot of this study. However, unfortunately, this study did not longitudinally compare the mental health data of college students during the preparation phase, the punctum maximun phase, and the return to normality phase, and such a study is important for revealing the natural changes in the psychological status of college students and their self-recovery ability during major public health emergencies. Another shortcoming is that the respondents in this study were mainly from college students, and the obtained findings may not be generalizable to other groups.

## Data availability statement

The raw data supporting the conclusions of this article will be made available by the authors, without undue reservation.

## Ethics statement

The studies involving human participants were reviewed and approved by Xi’an Polytechnic University. The subjects provided their written informed consent to participate in this study. The patients/participants provided their written informed consent to participate in this study.

## Author contributions

ZW analyzed the experiments and wrote the manuscript. HX conceived and coordinated the study. XW designed the experiments and participated in questionnaire design. BJ performed charts and figures. YN carried out data collection. All authors reviewed the results and approved the final version of the manuscript.

## Conflict of interest

The authors declare that the research was conducted in the absence of any commercial or financial relationships that could be construed as a potential conflict of interest.

## Publisher’s note

All claims expressed in this article are solely those of the authors and do not necessarily represent those of their affiliated organizations, or those of the publisher, the editors and the reviewers. Any product that may be evaluated in this article, or claim that may be made by its manufacturer, is not guaranteed or endorsed by the publisher.
